# Genome-Wide Association Study and Genomic Prediction for Bacterial Wilt Resistance in Common Bean (*Phaseolus vulgaris*) Core Collection

**DOI:** 10.3389/fgene.2022.853114

**Published:** 2022-05-31

**Authors:** Bazgha Zia, Ainong Shi, Dotun Olaoye, Haizheng Xiong, Waltram Ravelombola, Paul Gepts, Howard F. Schwartz, Mark A. Brick, Kristen Otto, Barry Ogg, Senyu Chen

**Affiliations:** ^1^ Department of Horticulture, University of Arkansas, Fayetteville, AR, United States; ^2^ Organic & Specialty Crop Breeding, Texas A&M AgriLife Research, Vernon, TX, United States; ^3^ Department of Plant Sciences/MS1, University of California, Davis, Davis, CA, United States; ^4^ Department of Agricultural Biology, Colorado State University, Fort Collins, CO, United States; ^5^ Department of Soil and Crop Sciences, Colorado State University, Fort Collins, CO, United States; ^6^ Department of Plant Pathology, University of Minnesota, Minneapolis, MN, United States

**Keywords:** common bean, bacterial wilt, genome-wide association study, genomic prediction, single nucleotide polymorphism, *Phaseolus vulgaris*, *Curtobacterium flaccumfaciens* pv. flaccumfaciens

## Abstract

Common bean (*Phaseolus vulgaris*) is one of the major legume crops cultivated worldwide. Bacterial wilt (BW) of common bean (*Curtobacterium flaccumfaciens pv. flaccumfaciens*), being a seed-borne disease, has been a challenge in common bean producing regions. A genome-wide association study (GWAS) was conducted to identify SNP markers associated with BW resistance in the USDA common bean core collection. A total of 168 accessions were evaluated for resistance against three different isolates of BW. Our study identified a total of 14 single nucleotide polymorphism (SNP) markers associated with the resistance to BW isolates 528, 557, and 597 using mixed linear models (MLMs) in BLINK, FarmCPU, GAPIT, and TASSEL 5. These SNPs were located on chromosomes *Phaseolus vulgaris* [Pv]02, Pv04, Pv08, and Pv09 for isolate 528; Pv07, Pv10, and Pv11 for isolate 557; and Pv04, Pv08, and Pv10 for isolate 597. The genomic prediction accuracy was assessed by utilizing seven GP models with 1) all the 4,568 SNPs and 2) the 14 SNP markers. The overall prediction accuracy (PA) ranged from 0.30 to 0.56 for resistance against the three BW isolates. A total of 14 candidate genes were discovered for BW resistance located on chromosomes Pv02, Pv04, Pv07, Pv08, and Pv09. This study revealed vital information for developing genetic resistance against the BW pathogen in common bean. Accordingly, the identified SNP markers and candidate genes can be utilized in common bean molecular breeding programs to develop novel resistant cultivars.

## Introduction

Common bean (*Phaseolus vulgaris L*.) is an important legume crop known for its edible seeds and pods worldwide (Allen 2013). It is an important source of protein for humans and livestock. Among legume crops, common bean is considered an outstanding source of nutrition and value in comparison to lentils (Ganesan and Xu 2017), fava beans (Juncus 1998), and chickpeas (Allen 2013). It is called the perfect food due to its content in protein (Guzmán-Maldonado et al., 2000), fiber (Hughes and Swanson 1989), and carbohydrates (Celmeli et al., 2018). It is mostly consumed as dry bean and green bean or snap bean in different parts of the world. On average, nearly 1.5 to 1.7 million acres of common bean is produced annually in the United States of America USDA-NASS Dry Beans (2022).

Common bean production has been affected by several seed-borne diseases (Sendi et al., 2020). Bacterial wilt (will be abbreviated as BW) of common bean caused by *Curtobacterium flaccumfaciens* pv. flaccumfaciens (Cff), affects production of common bean in a major way due to its seed-borne nature and is caused by various isolates of Cff (González et al., 2005). The pathogen is known to primarily cause disease in legume crops such as cowpea (*Vigna unguiculata*), common bean (*Phaseolus vulgaris*), mung bean (*Vigna radiata*), pea (*Pisum sativum*), and soybean (*Glycine max*) (Osdaghi et al., 2020). BW was first reported in South Dakota in 1926 (Hedges, 1926) and was later discovered in Mexico (Yerkes and Crispin, 1956), Canada (Hsieh et al., 2003), and in several other parts of the world. Due to its virulent nature and economic impact on legume crops, it is considered a high-risk pathogen that is subjected to quarantine regulations in Europe (CABI and Eppo, 1996).

The disease is transmitted *via* infected seeds (Hsieh et al., 2006). The *Cff* pathogen has five different isolates based on different color variants, that is, orange, yellow, purple, red, and pink (González et al., 2005). The infected seeds specifically turn to the color variant 3: making them yellow, orange, or purple as the infection proceeds. The disease symptoms include chlorotic areas on leaves with necrosis leading to a yellow halo progressing to irreversible plant wilt (Harveson et al., 2011). The leaf wilting is accompanied by hindrance of the normal water movement within the plant vascular system (Huang et al., 2009). The symptoms are worsened to tearing and shredding of leaves under unfavorable weather conditions. Young seedlings and plants are more susceptible to disease and prone to early mortality than mature plants (Hsieh et al., 2006; Osdaghi et al., 2020). The disease occurrence is primarily attributed to seed discoloration as a common symptom of BW in common bean (Hsieh et al., 2006). Mature seeds of infected plants are discolored and show yellow, orange, or purple seed coats (Harveson et al., 2011).

BW causes economic losses due to a substantial decrease in crop yield and marketability of the grain produced due to the visual appearance, size, shape, and color of the infected seeds (Huang et al., 2009). Crop rotation coupled with the use of pathogen-free seeds has been used to control the disease (Harveson et al., 2011). However, a cost-effective and reliable measure for disease management is to explore genetic resources to develop resistant cultivars (Assefa et al., 2019). Limited research has been conducted for BW management in common bean (Assefa et al., 2019). Early studies, based on a segregating population resulting from a cross between a resistant and susceptible genotype, identified the susceptibility to BW to be governed by two complimentary dominant genes. However the inheritance pattern for resistance was not clearly determined (Coyne et al., 1965). A more recent study identified a genotype showing some degree of resistance through inoculation tests, but it required substantial level of backcrossing to be acceptable for open cultivation in farmer’s fields (Urrea and Harveson 2014).

More recently, resistant cultivars such as the great northern bean “Resolute,” pinto bean “Agrinto,” pink bean “Early Rose” (Mündel et al., 2005), and an advanced black bean line L02F132 have been identified in Canada, which are resistant to three isolates of *Cff* (Mündel et al., 2005). The bean breeding program at Alberta has also evaluated the identified resistant lines for resistance against different diseases of common bean (Zienkiewicz., 2016). Limited research has been conducted in the United States, resulting in the development of a tolerant variety, namely, great northern cv. “Emerson” (Coyne et al., 1971) as the first cultivar, tolerant to three isolates of the BW pathogen, which was derived by pedigree selection between a BW resistant genotype and the great northern bean type. However, under hot dry field conditions, the symptoms of BW were again observed at early stages of plant growth for this tolerant cultivar (Coyne et al., 1971). Moreover, the resurgence of BW, specifically in Nebraska, suggests there is a need to conduct comprehensive studies to identify genetic resistance to this pathogen (Huang et al., 2009).

Evaluating the existing bean germplasm for the identification of resistance to BW is vital and a cost-efficient method of disease management. BW resistant bean cultivars can be a useful resource in worldwide common bean breeding programs. The identified new sources of resistance to *Cff* will enable breeders to develop reliably resistant cultivars for the future. The source of genetic resistance identified in common bean commercial cultivars can also be transferred to susceptible, elite cultivars through conventional breeding to enhance sources of resistance.

Molecular breeding in plants has played a vital role for crop improvement by expediting crop breeding through the use of molecular tools (Mammadov et al., 2012; Id, Id, and Mayer 2018). Major genes and alleles have been tagged to facilitate marker-assisted selection (MAS) (Heffner et al., 2009; Assefa et al., 2019; Larkin et al., 2019). Recently, genomic selection (GS) has emerged as a valuable tool for crop improvement through predictive breeding (Visscher et al., 2012; Chung et al., 2017; Keller et al., 2020). GS employs the use of genomic estimated breeding value (GEBV) to select individuals based on their performance and has been successfully employed in the breeding programs for crops such as soybean (Jarquin et al., 2016; Qin et al., 2019; Ravelombola et al., 2019; Wen et al., 2019), maize (Liu et al., 2019; Cui et al., 2020), rice (Spindel et al., 2015; Wang et al., 2020), and wheat (Larkin et al., 2019) targeting disease resistance (Heffner et al., 2009; Vallejo et al., 2017; Carpenter et al., 2018) and other important agronomic traits (Chung et al., 2017). Similarly, GS and GWAS have been deployed to study environmental stresses affecting important agronomic traits in common bean (López-Hernández and Cortés, 2019; Keller et al., 2020; Delfini et al., 2021). However, no published GWAS studies have been reported in common bean that specifically address resistance to BW.

Historically, SNP genetic maps have been constructed in common bean using 6K SNP BeadChips (Santos et al., 2003). The availability of several genome assemblies of common bean (e.g., Schmutz et al., 2014; Vlasova et al., 2016; Rendón-Anaya et al., 2017) has helped breeders conduct SNP studies for different traits allowing identification of candidate genes for important agronomic traits such as drought tolerance (Villordo-Pineda et al., 2015; Valdisser et al., 2020).

Hence, GWAS and GS serve as valuable tools for genetic improvement of important traits in crop species (Chung et al., 2017). The reduced cost of genotyping and improved methods of statistical analysis have increased the availability of valuable genetic information in large populations for complex traits (Visscher et al., 2012). Accordingly, this study primarily focuses on the evaluation of BW resistance in a publicly available USDA common bean core collection (Kuzay et al., 2020; Shi et al., 2021), using association mapping to identify SNP markers associated with BW resistance and conduct GS with the associated SNPs followed by candidate gene discovery.

## Materials and Methods

### Plant Materials

A subset of 168 accessions from the USDA common bean core collection was used in this study. Around 50% (85 accessions) of the total accessions were collected from Mexico. The remaining 83 accessions were collected from Guatemala (20), Colombia (18), Costa Rica (10), Nicaragua (10), Ecuador (9), El Salvador (5), Honduras (4), and Peru (7) (Supplementary Table S1).

### Bacterial Wilt Isolates

Three isolates of BW were used to study resistance to the *Cff* pathogen in this study. The yellow (BW_528), orange (BW_557), and purple (BW_597) isolates of the BW pathogen were previously recovered and maintained from infected common beans in Nebraska (Harveson et al., 2011) or Colorado. The purple isolate was obtained from the collection of R. Harveson at the University of Nebraska (Harveson et al., 2011), and the other two isolates were obtained from the collection of H. Schwartz (Schwartz et al., 2009) at Colorado State University.

### Phenotyping for Bacterial Wilt Resistance

The 168 common bean accessions were tested for the three isolates of BW. The experiment was conducted using the cotyledonary node inoculation method (Hsieh et al., 2003) by Howard F. Schwartz at Colorado State University and Mark A. Brick, Kristen Otto, and Barry Ogg at Colorado State University (Schwartz et al., 2010). The data set with disease scores is already published and available for public at the USDA GRIN website (https://www.ars-grin.gov/Pages/Collections).

In brief, 7–8 seeds were sown at a depth of 2.5 cm using 15 cm plastic pots with a standard potting mix. The seedlings were thinned to five plants upon emergence. The 7- to 10-day-old seedlings were inoculated with the respective isolate using a sterile needle. The inoculated seedlings were then incubated at a daily temperature of 28°C/22°C for 16 h per day and 8 h per night photoperiod in a greenhouse. A total of 9–12 plants per accession were used for evaluation for each isolate. In addition, ten plants for the resistant and susceptible controls were included for each BW isolate. The symptoms were evaluated 4 weeks post inoculations. Data were recorded as average severity for the replicated plants for each isolate. A 2-month cycle was used to evaluate the germplasm for each isolate individually.

A standard rating scale from 1 to 4 was used to evaluate the plants, with 1 as highly resistant demonstrating no wilt or discoloration, 2 being moderately resistant with wilt or discoloration at one of the unifoliolate leaves, 3 showing wilt or discoloration on both unifoliolate leaves but asymptomatic on the 1st trifoliolate leaf, and 4 as highly susceptible with wilt or discoloration on the 1st trifoliolate leaf (Schwartz et al., 2009).

### Genotyping

The common bean core set was genotyped (Kuzay et al., 2020) using BARCBean6K_3 Infinium BeadChips (Song et al., 2013). A total of 4,568 SNPs were obtained from the BARCBean6K_3 Infinium BeadChips (https://datadryad.org/stash/dataset/doi:10.25338/B8KP45) for genotyping. SNP filtering was conducted with removal of SNPs; data missing rate >20%, heterogeneous >10%, and MAF (minor allele frequency) <5%.

### Phenotypic Data Analysis and Estimation of Plant Distribution for Bacterial Wilt Isolates

The phenotypic data for the three BW isolates resistance was analyzed using ANOVA and GLM functions in JMP Genomics 7 (Cary 2008). The mean (X), variance (V), standard deviation (SD), and standard error (SE) were estimated using the “Tabulate” function in JMP Genomics 7 followed by the “Distribution” function to graphically present the phenotypic data for each of the BW isolates.

### Estimation of Population Structure and Genetic Diversity

The principal component analysis (PCA) and genetic diversity were analyzed using GAPIT 3 (genomic association and prediction integrated tool version 3) by setting PCA = 2 to 10 and NJ tree = 2 to 10, and phylogenetic trees were drawn using the neighbor-joining (NJ) method (Lipka et al., 2012; Wang et al., 2021; https://github.com/jiabowang/GAPIT3).

### Association Analysis

The phenotypic and genotypic data obtained for the 168 common bean core collection was subjected to genome-wide association mapping using the mixed linear model (MLM) methods in TASSEL 5 (Bradbury et al., 2007). The compressed mixed linear modeling (cMLM) in GAPIT (Lipka et al., 2012), FarmCPU (Liu et al., 2016), and Bayesian-information and Linkage-disequilibrium Iteratively Nested Keyway (BLINK) (Huang et al., 2019) were performed using the GAPIT 3 tool (Lipka et al., 2012; Wang et al., 2021; https://zzlab.net/GAPIT/index.html; https://github.com/jiabowang/GAPIT3). A threshold LOD [log10(p)] value >3.0 was used to select significant SNP markers associated with resistance to the BW_528, BW_557, and BW_597 isolates. Squared correlation coefficient (R^2^) was used to calculate the linkage disequilibrium (LD) between the markers.

### Candidate Gene Prediction

The SNP regions were subjected to candidate gene discovery analysis for the identification of candidate genes spanning the 50 kb (50 kb on each side of SNP) regions around the significant SNPs. The Andean whole-genome reference sequence *Pvulgaris* 442_v2.1 available on the Phytozome website (https://phytozome.jgi.doe.gov/pz/portal.html) was used to retrieve the candidate genes from the reference annotation of the common bean genome.

### Genomic Prediction for Genomic Selection of Bacterial Wilt Resistance

In addition to the identification of SNPs associated with BW resistance, the effect of these SNPs markers was also evaluated by using seven genomic prediction (GP) models. The ridge regression (RR); best linear unbiased prediction (BLUP) rrBLUP analysis; Bayesian models: Bayes A, Bayes B, Bayes ridge regression (BRR), and Bayes LASSO (BL); random forest (RF); and support vector machines (SVM) were deployed to assess GP (Table 3).

GP were carried out using the unbiased prediction in the rrBLUP (Wang J. et al., 2018) package to predict for GS utilizing the GEBV (Vallejo et al., 2017) with the R software version 3.5.0 (https://www.r-project.org). The Pearson correlation coefficient (r) was used to estimate the prediction accuracy (PA) using the GEBVs and observed values (Waldmann 2019) for the resistance to each of the three BW isolates. In addition, Bayesian models: Bayes A, Bayes B (Barili et al., 2018), Bayes ridge regression (BRR), and Bayes LASSO (BL) (Legarra et al., 2011); random forest (RF) (Ogutu et al., 2011); and support vector machines (SVM) (Maenhout et al., 2007) were deployed to assess the GP. Each combination of GP was run hundred times to estimate the GP statistical parameters, including variance (V), mean (X), standard deviation (SD), standard error (SE), and (r) values. Two approaches were used in combination with the seven prediction models 1) using all the 4,568 SNPs and 2) using the 14 selected SNP markers. The distribution plots were drawn using the R package ggplot2 and Microsoft Excel 2016.

## Results

### Phenotypic Data Analysis and Plant Distribution for Bacterial Wilt Isolates

**FIGURE 1:**
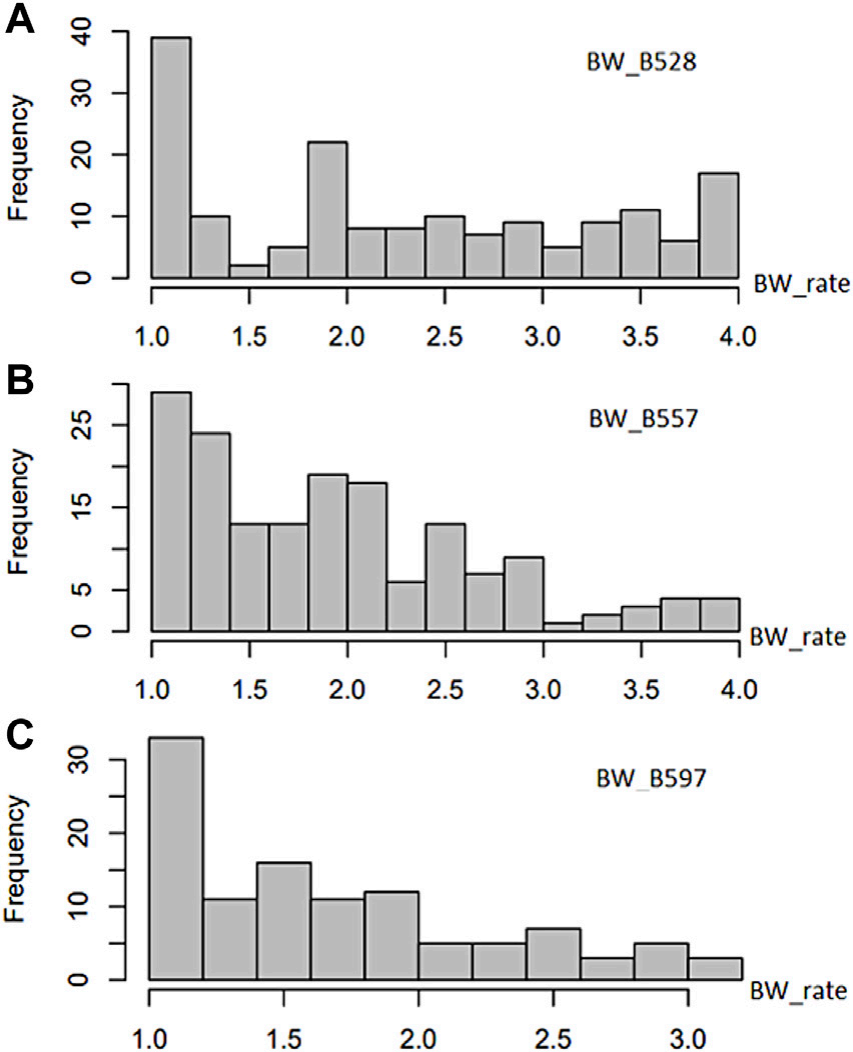
Distribution of bacterial wilt (BW) disease scale (0–4 rate) in 168 USDA common bean germplasm accessions.

The common bean core collection assessed for resistance to the three BW isolates showed a distribution of accessions from BW score 1 to 4 (where 1 is highest resistance and 4 is highest susceptible) skewed to resistance (Supplementary Table S1; Figure 1) to each of the three tested BW isolates, suggesting that BW resistant common bean accessions existed. Among the 168 common bean accessions inoculated with isolate BW_528, 31 accessions were resistant with a score of 1, while 9 accessions were susceptible with a score of 4. The distribution accessions for resistance to isolate BW_528 had a mean value (x) of 2.29, variance (V) of 1.027, standard deviation (SD) of 1.013, standard error (SE) of 0.078, and a coefficient of variance (CV) of 44.2% (Supplementary Table S2).

The distribution of resistance scores after inoculation of 165 accessions with the BW_557 isolate was skewed toward the left side with score 1 of BW resistance (Figure 1). Seven accessions were rated as highly resistant to BW_557 with a score of 1, while three accessions were scored as 4. The ANOVA analysis indicated a mean of 1.94 and an SD of 0.756 (Supplementary Table S2). Similarly, the graph for BW_597 was also skewed toward the left side. Among the 111 tested common bean accessions, nine accessions were rated 1 for resistance and one common bean accession imparted the highest susceptibility with a score of 3.17 (Supplementary Table S1; Figure 1). Overall, the distribution had a mean of 1.70, a median of 1.58, and an SD of 0.6 (Supplementary Table S2).

Based on the phenotypic analysis, PI203958 may be a good candidate for resistance to all three BW isolates. PI310611 was resistant to two BW isolates (BW_528 and BW_557) (Supplementary Table S1). Similarly, PI207336, PI313429, PI313531, PI325685, and PI451889 were potential candidates for resistance to the BW_528 and BW_597 isolates (Supplementary Table S1).

### Genetic Diversity and Population Structure

**FIGURE 2:**
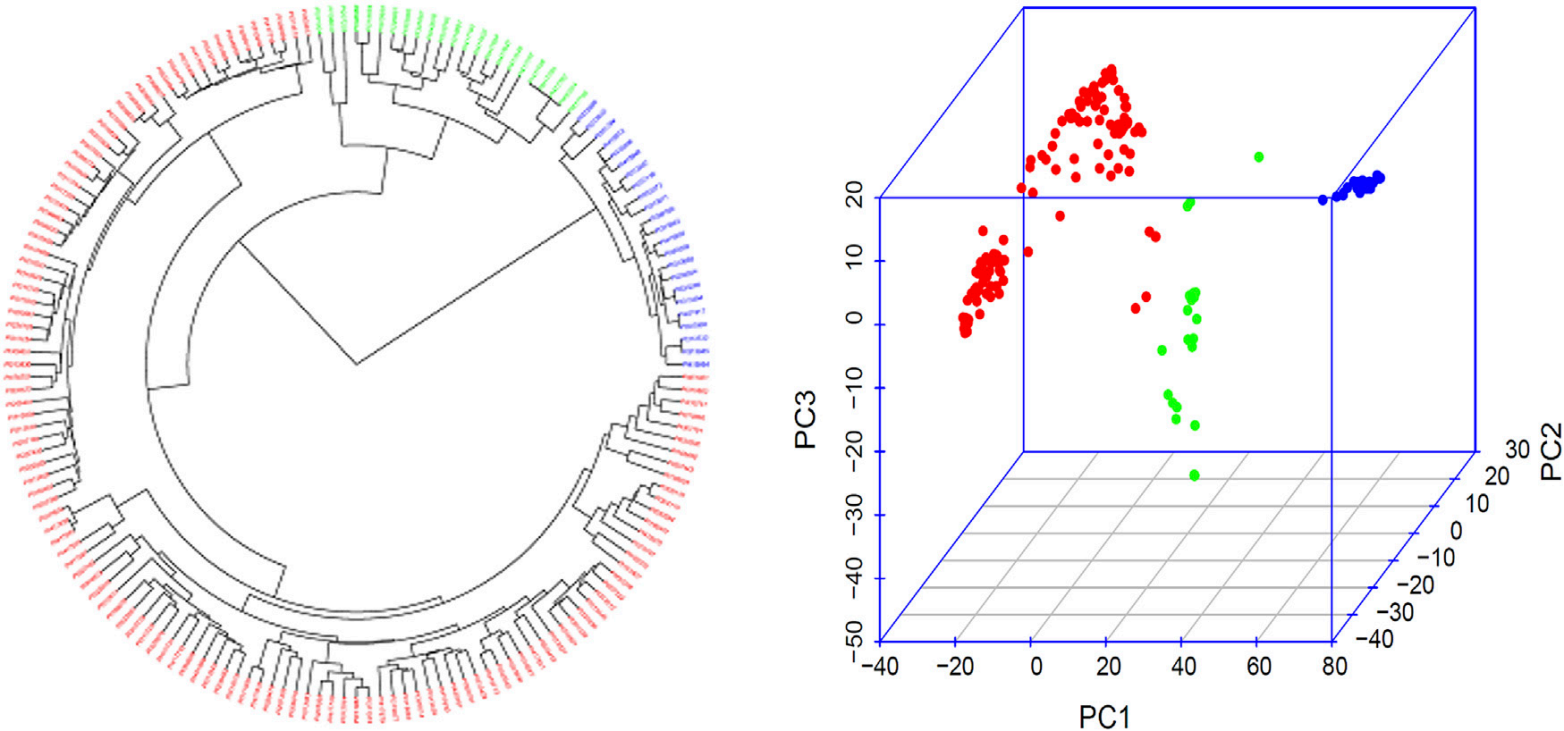
Population genetic diversity analysis in the association panel consisted of 168 USDA common bean germplasm accessions. Phylogenetic trees drawn by using the neighbor-joining (NJ) method in three subpopulation (left) and 3D graphical plot of the principal component analysis (PCA) (right) drawn by using GAPIT 3. A large phylogenetic tree of the three subpopulation for each of the 168 common bean accessions is shown in Supplementary Table S1.

The three subpopulations (Q1, Q2, and Q3) were well-differentiated with red, green, and blue colors (Figure 2; Supplementary Figure S1) in 168 common bean accessions based on 4,568 high-quality SNPs analyzed by GAPIT 3. From a total of 121 genotypes 72% of the total population was accountable for the cluster 1 (Q1); 13% of the total genotypes comprises 22 genotypes that made up the second cluster (Q2), and the remaining 22 genotypes makes up 13% of the total population and the third cluster (Q3) (Figure 2; Supplementary Figure S1).

### Genetic Diversity of Bacterial Wilt Resistant Lines

**FIGURE 3:**
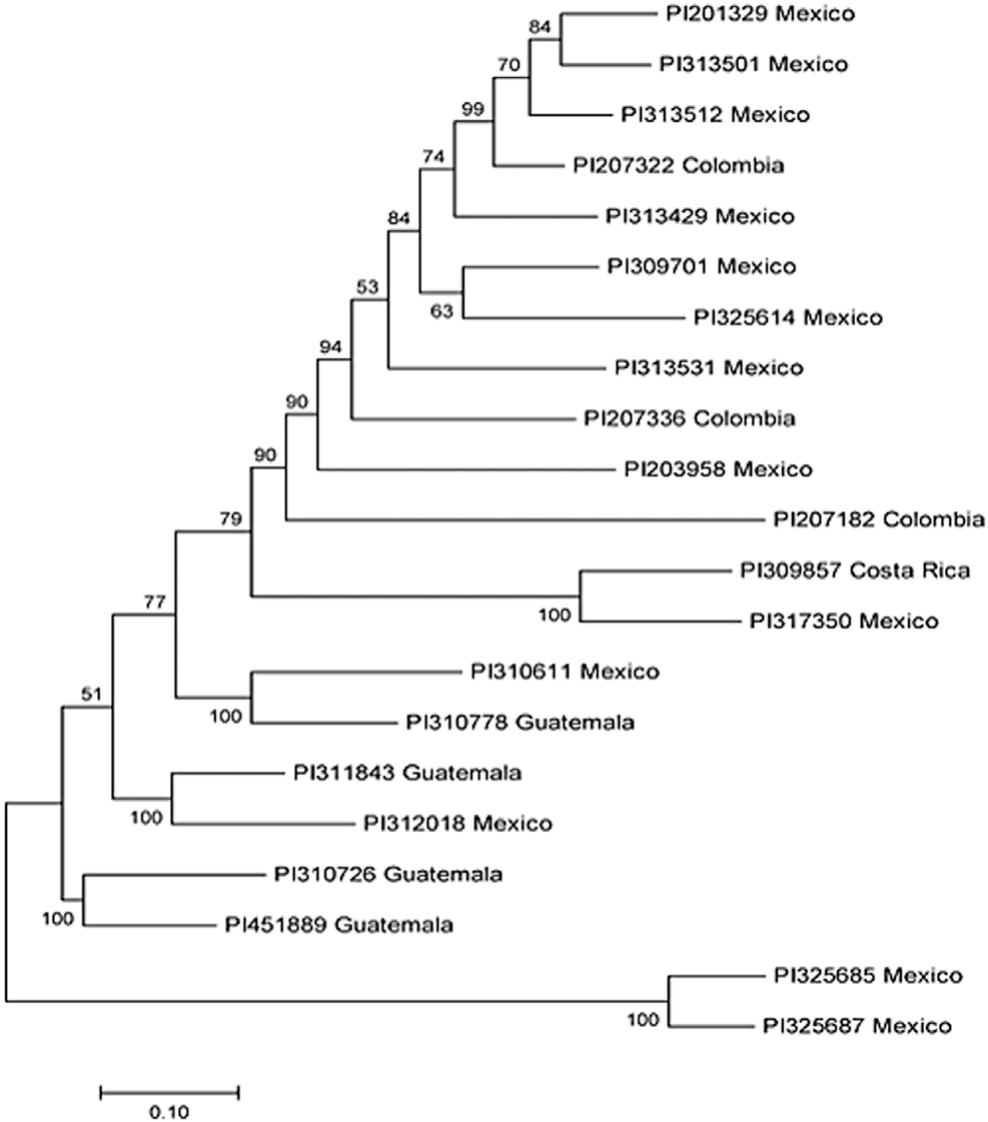
Phylogenetic tree among 21 common bean accessions of bacterial wilt resistance drawn using Mega 7. In the tree, the taxon name consists of the accession ID and the accession original country.

Our analysis identified a total of 21 R-lines (Table 1). The identified R-lines for all the three BW type were assigned to Q1 and Q2 clusters. Among the 21 R lines, 17 R-lines originated from Mexico, two from Colombia, one from Costa Rica, and one from Guatemala (Table 1). Here again, PI203958 from the Q1 subpopulation proved to be a good candidate with resistance to all the three BW isolates (Table 1). Furthermore, the phylogenetic tree also depicted a similar trend (Figure 3).

**TABLE 1:** List of 21 common bean accessions with resistance to three bacterial wilt (BW) isolates, B528, B557, and B597.

Campaign plant ID	Plant name	Country	Cluster	B528	B557	B597
PI207182	G918	Colombia	Q1	1	1.25	1
PI207322	Hidalgo 48-A	Colombia	Q1	1	1.08	1.08
PI207336	Jalisco 31-1	Costa Rica	Q1	1	1.09	1
PI309857	Col. No. 20670, lot #13	Guatemala	Q1	1	1.33	1.08
PI310726	Xucu mama	Mexico	Q1	1.17	1.36	1
PI310778	G2031	Mexico	Q1	1	1.08	1.25
PI311843	Frijol de gato	Mexico	Q1	1.08	1.17	1.25
PI451889	—	Mexico	Q1	1	1.17	1
PI201329	No. 3194	Mexico	Q1	1	1.08	—
PI203958	Negro	Mexico	Q1	1	1	1
PI309701	Frijol rosita	Mexico	Q1	1.08	1.08	1.17
PI310611	Frijol de bara	Mexico	Q1	1	1	1.17
PI312018	Frijol negro bolito	Mexico	Q1	1	1.08	1.17
PI313429	Morado claro	Mexico	Q1	1	1.17	1
PI313501	Parraleno colorado	Mexico	Q1	1	1.25	1.08
PI313512	Amarillo	Mexico	Q1	1.25	1	1.17
PI313531	Apetito	Mexico	Q1	1	1.17	1
PI317350	Frijol de raton	Mexico	Q1	1	1.08	1.09
PI325614	G16396	Mexico	Q1	1	1.17	1.17
PI325685	G12879	Mexico	Q2	1	—	1
PI325687	Frijol del raton	Mexico	Q2	1	1.08	1.18

### GWAS and SNP Marker Identification

Collectively, 14 SNPs were associated with resistance to BW_528, BW_557, and BW_597, respectively, based on the four MLM models in TASSEL 5, FarmCPU, GAPIT, and BLINK using the 4,568 SNPs (Table 2). The identified SNPs were associated with only single isolate, respectively, and not a SNP marker was simultaneously associated with all three isolates with an LOD value >3.0 for one or more of the four MLM models for resistance to all three isolates (Table 2). A total of 4,568 SNPs were used to conduct LD analysis. The LD decay started at around 137 kb (Supplementary Figure S2).

**TABLE 2:** List of the selected SNP markers associated with resistance to three bacterial wilt (BW) isolates B528, B557, and B598 in common bean core collection obtained from four MLM models in Tassel 5, BLINK, GAPIT, and FarmCPU and a *t*-test.

SNP	Chr	Pos	LOD[-log(P-value)]					R^2^%	Beneficial allele (resistant)	Unbeneficial allele (susceptible)	MAF%	BW strain
			Tassel_MLM	Blink	Gapit	FarmCPU	t-test	Tassel_MLM				
ss715647803	2	3915879	2.77	2.44	3.20	2.44	4.72	7.86	G	T	7.2	BW_528
ss715640165	4	12907955	2.17	3.24	2.72	3.24	10.93	6.12	C	T	7.8	
ss715648247	4	38819373	2.97	3.47	2.97	3.47	0.93	6.54	T	C	10.1	
ss715648541	8	12268429	2.74	4.26	3.12	4.26	6.99	7.81	T	G	37.2	
ss715639596	9	31079880	2.47	3.12	2.15	3.12	0.85	5.44	G	A	19.9	
ss715647928	7	11939824	2.37	3.03	2.77	3.03	1.02	5.34	G	A	3.0	BW_557
ss715648425	7	14455236	2.82	3.04	2.82	3.04	3.29	4.93	T	C	4.8	
ss715642582	7	14750979	2.82	3.04	2.82	3.04	3.29	4.93	G	T	4.8	
ss715648754	10	3784843	1.79	3.15	2.95	3.15	1.61	4.49	G	T	26.2	
ss715646271	11	2884160	3.01	3.59	3.18	3.59	2.26	6.88	T	C	3.6	
ss715649344	4	43584074	3.07	2.91	2.99	2.91	6.49	10.27	T	G	46.1	BW_597
ss715647896	8	42837392	3.04	2.84	3.09	2.84	17.05	10.16	G	A	6.5	
ss715641991	8	45046851	3.06	2.58	2.89	2.57	16.96	10.25	A	G	6.6	
ss715649486	10	8067409	2.39	3.16	3.04	3.15	4.07	10.12	T	C	36.4	

The *t*-tests for the 14 SNP markers are listed in Table 2, showing their allelic association with the phenotypes in each of the three BW isolates. Except ss715648247, ss715639596, and ss715647928, 11 markers had a LOD value >1.6, showing significant differences between two alleles of the 11 SNPs at a p-value at the 0.05 level (Table 2). Highly significant difference with a LOD >4.0 were observed at the three SNPs, ss715647803, ss715640165, and ss715648541, for BW_528 resistance and at the four SNPs, ss715649344, ss715647896, ss715641991, and ss715649486, for BW_597 resistance (Table 2), suggesting that the presence of beneficial alleles associated with BW resistance.

### GWAS for Bacterial Wilt_528 Isolate Resistance

The GWAS panel for BW_528 was subjected to four MLM analyses in TASSEL 5; a QQ-plot distribution was obtained for the observed vs. expected LOD values. Based on MLM, the distribution of QQ-plot between the observed vs. expected LOD value showed divergence from the expected distribution (Supplementary Figure S3). A similar trend was observed for the MLM QQ-plot with GAPIT, FarmCPU, and BLINK (Supplementary Figure S3). The QQ-plots obtained from GAPIT, BLINK, and FarmCPU showed the beginning of divergence between the observed vs. expected values starting at LOD >2 (Supplementary Figure S3).

These findings indicate the presence of SNPs at LOD scores greater than two to be associated with resistance to the BW_528 isolates (Supplementary Figure S3).

The TASSEL analysis showed a Manhattan plot for the MLM model with only one significant SNP (ss715648247 on chromosome Pv (04) had LOD close to 3 (actual value of 2.97) and other four SNPs with LOD >2.0 on Pv02, Pv04, Pv08, and Pv09, respectively, indicating a weak association for resistance to the BW_528 (Supplementary Figure S3). On the other hand, the cMLM model in GAPIT showed a Manhattan plot with significant SNPs (LOD values >3) for resistance on Pv02 and Pv08 (Supplementary Figure S3). The Manhattan plot from BLINK also showed a similar trend with associated SNPs (LOD values >3) located on chromosomes Pv04, Pv08, and Pv09 (Supplementary Figure S3). Likewise, the Manhattan plot obtained from FarmCPU also showed similar results as BLINK with the associated SNPs located on chromosomes Pv02, Pv04, Pv08, and Pv09 (Table 2; Supplementary Figure S3).

The combined results from all the four models of MLM in GAPIT, MLM in FarmCPU, BLINK, and TASSEL showed a total of five SNPs associated with resistance to the BW_528 isolate (Table 2). The two SNPs, ss715640165 and ss715648247, were positioned at 12,907,955 and 38,819,373 bp, respectively, on Pv0 4 (Table 2), while another SNP, ss715647803, located on Pv02 was positioned at 3,915,879 bp. The SNP markers, ss715639596 and ss715648541, were positioned at 31,079,880 bp on Pv09 and 12,268,429 bp on Pv08, respectively (Table 2).

### GWAS for Bacterial Wilt_557 Isolate Resistance

Based on MLM in TASSEL 5, the distribution of QQ-plot between the observed vs. expected LOD values showed divergence from the expected distribution. A similar trend was observed for the MLM QQ-plot obtained from the cMLM analysis from GAPIT and the MLM analysis from FarmCPU, and BLINK. The QQ-plots obtained from GAPIT, BLINK, and FarmCPU showed a larger divergence between the observed vs. expected values at LOD >2.5 (Supplementary Figure S4). This indicates the presence of SNPs at LOD >2.5 to be associated with resistance to the BW isolate 557 (Supplementary Figure S4).

The TASSEL analysis showed the Manhattan plot for the MLM model with SNPs associated with BW_550 resistance, being indicated as dots with LOD value greater than 3 to be located on chromosome Pv11 (Supplementary Figure S4). On the other hand, the MLM model resulted in a Manhattan plot with significant SNPs (LOD values >3) for resistance on Pv11 and Pv10 (Supplementary Figure S4). The Manhattan plot from BLINK also showed a similar trend with associated SNPs (LOD value >3) on Pv07, Pv10, and Pv11 (Supplementary Figure S4). The Manhattan plot obtained from FarmCPU showed similar results with the associated SNPs on Pv7, Pv10, and Pv11. However, no SNP was found to be associated with a LOD higher than 5.5 (Supplementary Figure S4).

The combined results from all the four models of MLM in GAPIT, FarmCPU, BLINK, and TASSEL showed a total of five SNPs associated with resistance to the BW_557 isolate. Three SNPs, ss715647928, ss715648425, and ss715642582, were located on chromosome Pv07 with the latter two located closely together on positions 14,455,236 and 14,750,979 bp, respectively (Table 2). The other two SNPs were located at position 3,784,843 bp on Pv10 and position 2,884,160 on Pv11, respectively (Table 2).

### GWAS for Bacterial Wilt_597 Isolate Resistance

The MLM QQ-plot distribution between the observed vs. expected LOD, obtained using the MLM in TASSEL 5, showed divergence from the expected distribution. A similar trend was observed for the MLM QQ-plot obtained from the cMLM analysis in GAPIT, and the MLM analysis in FarmCPU and BLINK (Supplementary Figure S5). The QQ-plots obtained from GAPIT, BLINK, and FarmCPU showed the beginning of divergence between the observed vs. expected values at LOD >2 (Supplementary Figure S5). This indicates the presence of SNPs at LOD >2 associated with resistance to the BW_597 isolate (Supplementary Figure S5).

The TASSEL analysis showed the Manhattan plot for the MLM model with only one SNP associated with resistance to the BW_597 isolate (LOD >3) located on chromosomes Pv04 and Pv08 (Supplementary Figure S5). The MLM model showed the Manhattan plot indicating associated SNPs (LOD values >3) for resistance on Pv04, Pv08, and Pv10 (Supplementary Figure S5). The Manhattan plot obtained from BLINK showed associated SNPs with a LOD >3 located on Pv02 and Pv10 (Supplementary Figure S5). The Manhattan plot obtained from FarmCPU showed associated SNPs located on Pv04, Pv08, and Pv10 (Supplementary Figure S5).

The combined results from all the four models of MLM in GAPIT, FarmCPU, BLINK, and TASSEL showed a total of four SNPs associated with resistance in common bean for the BW_597 isolate (Table 2). The two SNPs, ss715647896 and ss715641991, were closely positioned at 42,837,392 and 45,046,851 on chromosome Pv08, respectively (Table 2), while other SNPs, ss715649344 and ss715649486, were located at position 43,584,074 on Pv04 and position 8,067,409 on Pv10, respectively (Table 2).

### Candidate Genes for Bacterial Wilt Resistance

The candidate gene discovery was carried out for 50 kb genomic regions upstream and downstream of the identified significant SNPs for each isolate. A total of 14 gene models were discovered 50 kb upstream and downstream of the identified SNP region on chromosomes Pv02, Pv04, Pv07, Pv08, and Pv09 (Supplementary Table S3). A total of six genes (*Phvul.002G041600*, *Phvul.004G076900*, *Phvul.004G119800*, *Phvul.008G107000*, *Phvul.009G210400*, and *Phvul.002G041000*) were identified as candidate genes for resistance to the BW_528 isolate. Out of these six genes, five genes (*Phvul.002G041600*, *Phvul.004G076900*, *Phvul.004G119800*, *Phvul.008G107000* and *Phvul.009G210400*) were located within the 50 kb region of the associated SNP (ss715647803, ss715640165, ss715648247, ss715648541, and ss715639596) region, while one gene model (*Phvul.002G041000*) included the SNP itself (Supplementary Table S3). These genes encoded the NAC domain–containing protein 87, XB3 ortholog 3, and duplicated homeodomain-like superfamily protein in *Arabidopsis thaliana*, while other identified gene models were unclassified (Supplementary Table S3).

Similarly, a total of four gene models, *Phvul.007G104800*, *Phvul.007G112100*, *Phvul.007G112900*, and *Phvul.007G104500*, were identified as candidate genes for resistance to the BW_557 isolate. Among these four gene models, three (*Phvul.007G104800*, *Phvul.007G112100*, and *Phvul.007G112900*) were located within the 50 kb distance of the identified SNPs (ss715647928, ss715648425, and ss715642582, respectively), while one gene model*, Phvul.007G104500* include the identified SNP (ss715647928). The *Phvul.007G112100* encoded a disease-associated Leucine-rich repeat protein kinase family protein positioned at 14,476,334 to 14,479,522 bp on chromosome Pv07 (Supplementary Table S3), while the other two gene models, *Phvul.007G104800* and *Phvul.007G104500*, encoded a cytochrome P450, family 77, subfamily A, polypeptide 4, and chlorophyllase, respectively.

A total of four gene models, *Phvul.004G154500*, *Phvul.008G166000*, *Phvul.004G154100*, and *Phvul.008G172000,* were presumed candidate genes for BW_597 resistance (Supplementary Table S3). These genes encoded a protein kinase superfamily protein, flavonol synthase 1, nuclease-related domain (NERD), and thioesterase superfamily protein, respectively. *Phvul.004G154500* and *Phvul.004G154100* were located on chromosome Pv04 and included the ss715649344 SNP. The other two genes (*Phvul.008G166000* and *Phvul.008G172000*) were located on chromosome Pv08, within the 50 kb region of ss715647896 and ss71564199 SNPs, respectively (Supplementary Table S3).

### Genomic Prediction for Resistance to Bacterial Wilt Isolates

The use of all the seven models with aforementioned two approaches predicted the overall GA between the observed values and GEBV for the BW_528 isolate to fluctuate between 0.51 and 0.58 when 1) all the 4,568 SNPs were used and between 0.40 and 0.53 when 2) using the 14 selected SNPs. Similarly, the seven models resulted in a range of 0.37–0.46 when 1) all 4,568 SNPs were used for BW_557 in comparison to the reduced range of 0.30–0.44 when 2) using the selected 14 SNPs. A slightly higher range of average “r” value from 0.41 to 0.47 and 0.43 to 0.52 were observed for BW_597 when 1) all the 4,568 SNPs and 2) the 14 selected SNPs were used, respectively, in combination with the seven GP models (Table 3; Supplementary Figures S6, S7). The results were also verified to be similar through cross-validation across the seven GP models (Supplementary Figures S6, S7).

**TABLE 3:** Genomic prediction of seven models for resistance to three bacterial wilt (BW) isolates in two SNP sets: 1) all 4,568 SNPs and 2) 14 SNP markers.

GP model	BW_528		BW_557		BW_597		Average (model)	SNP set
	rȲ100	SE	rȲ100	SE	rȲ100	SE		
rrBLUP	0.56	0.012	0.45	0.012	0.45	0.016	0.49	all 4568 SNPs
BL	0.58	0.010	0.46	0.013	0.43	0.017	0.49	
BA	0.55	0.013	0.46	0.013	0.47	0.016	0.49	
BB	0.55	0.010	0.44	0.013	0.45	0.016	0.48	
BRR	0.56	0.011	0.45	0.013	0.43	0.016	0.48	
SVM	0.54	0.013	0.47	0.013	0.44	0.018	0.48	
RF	0.51	0.012	0.37	0.015	0.41	0.016	0.43	
Average	0.55	—	0.44	—	0.44	—	0.48	
rrBLUP	0.40	0.012	0.30	0.016	0.43	0.015	0.38	14 SNP markers
BL	0.51	0.011	0.41	0.012	0.51	0.015	0.48	
BA	0.53	0.011	0.41	0.009	0.52	0.013	0.49	
BB	0.53	0.010	0.39	0.013	0.52	0.015	0.48	
BRR	0.52	0.011	0.44	0.010	0.51	0.015	0.49	
SVM	0.49	0.013	0.39	0.012	0.44	0.015	0.44	
RF	0.48	0.012	0.43	0.011	0.49	0.016	0.47	
Average	0.49	—	0.40	—	0.49	—	0.46	

The general trend of PA was higher when a greater number of SNPs (4,568 SNPs) was utilized in combination with the seven models in comparison to the use of 14 selected SNPs for BW_528 and BW_557. However, observing the PA for individual models, the RF model indicated slightly higher PA when 14 SNPs set was used for BW_557 resistance. Conversely, the PA followed a general trend of higher range for the average values of (r) and for each of the individual models when using a lower number of SNPs (14 selected SNPs) (Table 3).

## Discussion

### Genetic Diversity and Population Structure for the Common Bean Germplasm

The population structure and genetic diversity analyses in this study indicated the presence of three subpopulations (Q1, Q2, and Q3) among the tested germplasm as examined by the tool GAPIT 3 (Figure 2; Supplementary Table S1). Historically, Andean and Mesoamerican pools are reported as two centers for common bean origin (Bitocchi et al., 2013; Gepts et al., 1986; Kwak and Gepts 2009; Mamidi et al., 2013). Our study also confirmed the existence of two gene pools by consistent appearance of accessions from Mexico, Guatemala, Nicaragua, El Salvador, Costa Rica, Honduras, Colombia, Ecuador, and Peru in our two subpopulation clusters, Q1 and G2 (Supplementary Table S1, Figure 2). Hence, we can conclude that our tested germplasm is composed of diverse accessions and belong to the original two gene pools.

### Genome Wide Association Study and SNP Marker Identification for Bacterial Wilt Resistance

The current study was focused on identifying SNP markers associated with resistance to the three isolates of BW in common beans. The phenotypic and genotypic data from the 168 accessions of the common bean core collection was subjected to the four MLM models in GAPIT, BLINK, FarmCPU, and TASSEL 5 to carry out GWAS analysis for each of the BW isolates. A total of 14 SNP markers were associated with resistance to the three different BW isolates, including five SNP markers for BW isolate 528, five SNP markers for BW isolate 557, and four SNP markers for BW isolate 597 (Table 2). These SNP markers were scattered on chromosomes Pv02, Pv04, Pv07, Pv08, Pv09, Pv10, and Pv11.

Genetic studies in BW studies have been primarily limited to other crops, such as extensive use of SSR (simple sequence repeat), AFLP (amplified fragment length polymorphism), and SCAR (sequence characterized amplified region) markers to map QTLs for BW resistance (Thoquet et al., 1996; Ashrafi et al., 2009; Wang et al., 2013) and generate a high-density genetic maps of inbred lines for BW resistance in tomato. The major QTLs were located on chromosomes 6 and 12 (Shin et al., 2020). Similarly, BW resistance has been explored using GWAS in peanut (*Arachis hypogaea L*.) with identification of four QTLs on chromosome 4 (Wang L. et al., 2018).

However, in common bean, studies have been limited to the use of genetic analysis for other diseases such as SCN. Jain et al. (2019) identified SCN resistance factors in common bean on chromosomes Pv04, Pv07, Pv09, and Pv11 based on the *Pvulgaris* v1.0_218 reference genome sequence (from Andean accession G19833) for various races (Jain et al., 2019). Likewise, our study also revealed that the resistance for BW for the three isolates is scattered on multiple chromosomes with the identification of SNPs on chromosomes Pv02, Pv04, Pv07, Pv08, Pv09, Pv010, and Pv011 (Table 2; Supplementary Figures S3–S5). Thus, our study is the first to report specifically the identification of SNPs associated with resistance to the BW isolates in common bean.

Similarly, a recent study was conducted to phenotype 467 accessions consisting of the NPGS core collection, 8 local, and 31 experimental lines from University of Nebraska for the orange BW isolate (Urrea and Harveson 2014). The results led to the identification of only one cultivar resistant to the tested BW isolate (Urrea and Harveson 2014). Likewise, our study has successfully led to the identification of potential SNPs for resistance to the BW isolates were identified, which can now be employed in marker-assisted selection to develop resistant cultivars. The identified SNPs for each BW isolate can be pyramided to develop a single cultivar with enhanced resistance to multiple isolates of BW.

### Candidate Genes

Our results indicate the presence of a putative *chlorophyllase* encoded as the gene model *Phvul.007G104500* in the SNP ss715647928 region (Supplementary Table S3). The ortholog of the *chlorophyllase* gene in Arabidopsis encoded by *AtCLH1* is found to be induced following tissue damage by a bacterial necrotrophic pathogen (Kariola et al., 2005). The downregulation of *AtCLH1* is linked to enhanced susceptibility to the necrotrophic pathogen, which showed its role as modulator of defense to various pathogens (Kariola et al., 2005). Our findings also suggest that the identified *chlorophyllase* gene can be a good candidate for resistance to BW_528. However, the *Phvul.007G104800* gene model encoding the cytochrome P450, family 77, subfamily A, and polypeptide 4 protein was found to be located 50 kb upstream and downstream of SNP ss715647928 associated with resistance to the BW_557 isolate (Supplementary Table S3). The two genes near the ss715647928 SNP are suitable candidates for BW_528 resistance.

Similarly, the upstream and downstream regions of SNPs ss715648425 and ss715642582 also comprised gene models *Phvul.007G112100* putatively encoding the leucine-rich repeat protein kinase family protein and an unclassified gene *Phvul.007G112900*, respectively (Supplementary Table S3).

Interestingly, the LRR domains have been explored as vital modulators of immunity in plant–pathogen interaction responses (Marone et al., 2013; Song et al., 2019). Song et al. (2019) reported a total of 348 NBS-LRR proteins and studied the loss of function characteristic of an LRR domain resulting in increased susceptibility to the BW pathogen in peanut (*Arachis hypogea*) (Song et al., 2019). Thus, the identified gene *Phvul.007G112100* for BW_557 isolate in our study can also be explored further as a source of resistance to the *Cff* pathogen in common bean.

Moreover, our study identified two additional genes, *Phvul.002G041000* and *Phvul.004G154100*, on chromosome Pv02 for BW_528 resistance associated with SNPs ss715647803 and ss715649344. The *Phvul.002G041000* gene encodes a nuclease-related domain (NERD)*,* associated with BW resistance to BW isolate 597 in common bean. Other reported genes (Supplementary Table S3) include the Arabidopsis NAC domain–containing protein 87, XB3 ortholog 3 in Arabidopsis, and duplicated homeodomain-like superfamily protein near the SNP regions for BW_528. The NAC genes play a vital role in plant immune responses by acting as regulators modulating the hypersensitive response and receptors of pathogen effectors in host plants. The identified SNP with the candidate gene *Phvul.004G076900* encoding the Arabidopsis *NAC* gene can be studied further to develop a deeper understanding of the respective gene as a potential modulator of immunity for the BW pathogen in common bean.

However, the genes associated with resistance to BW_597 encoded putative protein kinase superfamily protein cytochrome P450, flavonol synthase 1, and thioesterase superfamily protein. These genes are associated with the mechanism of wilting in several plants. Reportedly, the cytochrome P450 is a major component of the underlying resistance molecular mechanism for verticillium wilt in cotton. The *flavanol synthase 1* gene has been reported to be a constituent of the flavonoid pathway, which are important regulators of biotic and abiotic stresses as an integral component of hormone signaling pathways, such as in Arabidopsis (Owens et al., 2008). However, its role as a modulator of plant–pathogen interaction is not clear yet. On the other hand, the thioesterase superfamily protein has been studied as an enhancer of drought tolerance in tobacco (Zhang et al., 2012), which makes it a suitable candidate as the modulator of abiotic stress tolerance. However, our study reported a SNP encoding the thioesterase superfamily protein. Based on our results, more studies should be conducted to ascertain the putative role of the respective genes for the common bean–*Cff* interaction.

So far, no other studies have been reported for candidate gene discovery for BW resistance in common bean. Our study, on the other hand, has successfully identified the presence of putative candidate genes associated with the BW resistance in common bean.

### Genomic Prediction for Genomic Selection

In this study, GP was conducted using two approaches: 1) by using all 4,568 SNPs and 2) by using the 14 selected SNPs, in combination with seven GP models 1) rrBLUP, 2) Bayes A, 3) Bayes B, 4) BL, 4) BRR, 5) RF, 6) SVM, and 7) BL for each of the three BW isolates.

The average (r) calculated for all the SNPs and the 14 SNPs (Table 3) indicated an overall lower value when the selected 14 SNPs were used (Table 3; Supplementary Figures S6, S7). However, exploring PA further using the entire seven models predicted slightly different trends for BW_557 and BW_597 in each of the two approaches. The BW_528 followed the similar trend of average (r) as obtained for the general PA. The BW_557 depicted slightly higher values of PA for the 14 SNPs set with the RF model (Table 3; Supplementary Figures S6, S7), deviating from the general trend previously obtained. In addition, the PA has been reported to be low with use of less number of SNPs (Ali et al., 2020). Using a SNP set of 2000 or more reportedly shows an r value of 0.85 in comparison to the r value of 0.80 when less SNPs (1000 SNPS) were used for a population for soybean accessions (Zhang et al., 2016). Likewise, our study also showed a similar trend for BW_528 and BW_557, suggesting that the use of a higher number of SNPs is more reliable for GP. On the other hand, BW_597 had a very different trend with a higher PA for the 14 selected SNPs set with the five models (Bayes A, Bayes B, BL, BRR, and RF) and with lower PA for the respective SNPs set for the rrBLUP and similar PA for the SVM model (Table 3; Supplementary Figures S6, S7). The different trends suggest that it might be more beneficial to perform GP using the 14 SNPs selected from the GWAS analysis for BW_597 rather than deploying the generic 4,568 SNPs, to estimate GP more accurately with the seven models for BW_597. Evidently, Qin et al. (2019) reported that the r values deviate from a higher range of 0.64–0.74 when GWAS-selected SNPs were deployed to carry out GP rather than using the randomly selected SNPs (Qin et al., 2019). Qin et al. (2019) also reported the average correlation coefficient (r) among the 15 amino acids to range from 0.18 to 0.61 when all the 23,279 SNPs were used for GP and 0.45 to 0.68 upon using 231 SNP markers, using the rrBLUP model (Qin et al., 2019). Accordingly, the trend for BW_597 in our study is justified and supports the use of 14 GWAS-derived SNPs in combination with the seven GP models to be more beneficial for genomic prediction rather than use of all the generic 4,568 SNPs (Table 3; Supplementary Figures S6, S7). The results confirmed the accuracy of using the predicted models accordingly, with similar results.

Moreover, GS, based on the estimation of PA through use of Pearson’s correlation coefficient (r) between the observed values and GEBV, has been employed to assess 481 common bean elite lines for resistance to environmental stress with reported prediction abilities between 0.6 and 0.8 for various traits (Keller et al., 2020). Several other studies have been reported for various biotic and abiotic stresses in common beans with genomic prediction (Barili et al., 2018; Jain et al., 2019; Shi et al., 2021), but no study has been reported to date for BW resistance in common bean utilizing genomic prediction. Significantly, here we report the use of genomic prediction for BW resistance in common bean, deploying seven prediction models under two SNP scenarios. The changes in the values of genomic prediction accuracies in our study indicate that the PA was affected by the SNP population size for our set of tested germplasm. Thus, our results indicate that the GS prediction can be effectively used in combination with MAS to breed for BW resistance in common bean.

## Conclusion

Our study successfully tested 168 common bean accessions from the USDA NPGS based on public phenotypic resistance data for genome-wide association study (GWAS) and genomic prediction (GP). A total of 14 SNPs, on chromosome Pv02, Pv04, Pv07, Pv08, Pv09, Pv010, and Pv011 with 14 candidate genes, and 21 lines with potential resistance to the BW_528, BW_557, and BW_597 types were identified as a result of this study. The different SNPs and candidate genes identified for each isolate can be pyramided to enhance resistance to multiple isolates of BW. Moreover, the identified SNPs and candidate genes can be further explored and employed using genome editing and breeding techniques to develop common bean cultivars with enhanced resistance to the three BW isolates.

## Data Availability

The original contributions presented in the study are included in the article/Supplementary Material, further inquiries can be directed to the corresponding authors.

## Supplementary Material

Data Sheet 1 Supplementary Figures

Data Sheet 2 Supplementary Tables
